# Pro-eating disorder search patterns: the possible influence of celebrity eating disorder stories in the media

**DOI:** 10.1186/s40337-016-0094-2

**Published:** 2016-03-03

**Authors:** Stephen P. Lewis, Laura Klauninger, Ivana Marcincinova

**Affiliations:** University of Guelph, Guelph, ON N1G 2W1 Canada

**Keywords:** Pro-eating disorder, Thinspiration, Celebrity, Website, Internet, Online

## Abstract

Pro eating disorder websites often contain celebrity-focused content (e.g., images) used as *thinspiration* to engage in unhealthy eating disorder behaviours. The current study was conducted to examine whether news media stories covering eating disorder disclosures of celebrities corresponded with increases in Internet searches for pro eating disorder material. Results indicated that search volumes for pro eating disorder terms spiked in the month immediately following such news coverage but only for particularly high-profile celebrities. Hence, there may be utility in providing recovery-oriented resources within the search results for pro-eating disorder Internet searches and within news stories of this nature.

Pro-eating disorder (pro-ED) websites are prevalent and typically involve individuals sharing strategies and messages that promote or encourage eating disorder (ED) behaviours; this includes sharing images of emaciated celebrities, which serve as “thinspiration” for extreme weight loss [1–3]. Researchers have documented that youth and emerging adults are the primary users of these websites and that exposure to pro-ED websites is associated with ED pathology [1–3].

One solution to mitigate the potential impact of pro-ED websites is to provide pro-recovery resources in the search results corresponding to pro-ED Internet searches [2, 4]. In this way, access to (and the potential impact of) pro-ED websites may be circumvented - or at least delayed. Similar strategies are currently in place for suicide web-searches on Google [5]. To do this effectively, however, it may be important to not just consider *what* pro-ED terms may be sought out on popular search engines but to also consider *fluctuations* in pro-ED web-searches [2, 4]; in particular, there may be merit in considering what might account for such changes [2]. To date, no study has examined what factors may influence pro-ED search patterns online.

As celebrities are used as sources of *thinspiration* [1–3], and a number of celebrities have publicly disclosed ED histories via news media [6], it is possible that public ED disclosures by celebrities represent one factor influencing pro-ED search patterns online. Findings from research examining media coverage of suicide add support for this possibility. Specifically, pursuant to media coverage of several suicides, and within the geographic region of the stories, increases in Google search patterns for the suicide methods reported in the stories have been documented [7].

Should stories covering celebrity ED disclosures impact pro-ED search patterns, it may be necessary to provide resources promoting ED prevention and recovery within people’s search results, before potentially harmful content is accessed. Accordingly, we conducted the present study to determine whether Internet searches for pro-ED keywords changed pursuant to dissemination of a celebrity ED disclosures covered by news media. To do this, we examined pro-ED search trends on Google, the most used search engine worldwide [8]. We anticipated that the timing of at least some news stories would correspond with increases in pro-ED searches soon after the story was published; furthermore, we expected that this would be especially the case for high-profile celebrities, who ostensibly have a larger following and thus a possible greater influence on others.

## Methods

We first identified high-profile media stories in which celebrities disclosed past ED experiences using Google News [6]. To index individual celebrity popularity, we consulted popular social networks. Specifically, we examined the number of *likes* these celebrities’ official pages received on Facebook and the number of followers they accumulated on Twitter. From these efforts, one celebrity was shown to have a significant following, namely Lady Gaga. Her likes on Facebook were approximately 25 million more, and she had over 15 million more Twitter followers, than the next celebrity on our list. Although we focused most of our attention on her story in our analysis, we also compared changes in pro-ED search patterns associated with her story with those of other celebrity stories involving public ED disclosures.

To determine whether there were changes in search patterns for pro-ED terms around the timing of news stories reporting Lady Gaga’s public ED disclosure, Google Trends was used [9]. After initially entering relevant keywords, this program allows users to generate frequency data for individual Google search terms by narrowing the results to particular time periods and locations. Past research has used this program to identify pro-ED search terms (e.g., *pro ana, pro thinspiration*) associated with particularly harmful pro-ED content [2]. We therefore used these search terms in the current study to determine whether the search volumes associated with each one changed when comparing the month before, the month of, and the month after news stories reported Lady Gaga’s ED history. We attenuated focus to searches in the United States for two reasons. First, Lady Gaga is from the United States, as were other celebrities we identified. Second, celebrities may be used as sources of *thinspiration* [1–3] and Google Search terms related to *thinspiration* may be especially sought out in the United States [2].

## Results

### Analyses

**Fig. 1:**
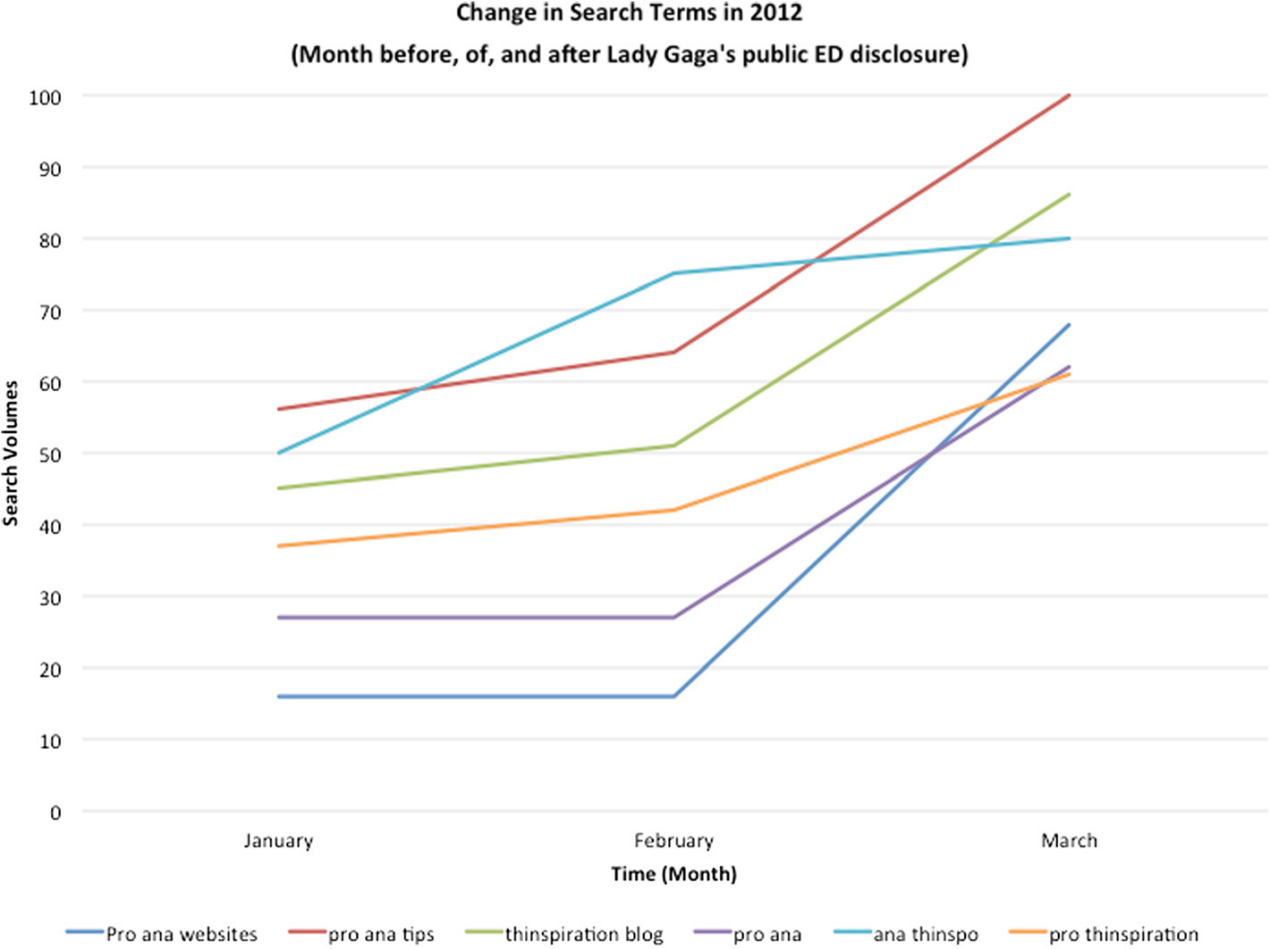
Search volumes for pro-ED key words via Google from January to March 2012. Notes: Values represent normalized rather than absolute values of the number of online pro-ED searches for the month before (January, 2012), at the time of (February, 2012), and after (March, 2012) Lady Gaga’s public ED disclosure via news media

Data from Google Trends pertaining to various search terms are normalized and scaled out of 100; they do not represent raw search volumes. For instance, if one term (e.g., Term X) received a value of 100 and another term (e.g., Term Y) had a value of 50, the latter (Term Y) would have received one half of the searches as the former (Term X) over a designated period of time. Upon obtaining search data for the above mentioned terms and following approaches used in other research using Google Trends to examine search term fluctuation following news stories [8], we compared search volumes for the terms noted above by examining the change (if any) between: a) the month prior to the news story, b) the month of the story and c) the month after the story was published. To do this, a series of paired t-tests^1^ were computed to determine if there were significant changes in pro-ED search patterns between the above months of interest. [7]. Changes in pro-ED search term volumes from January to March of 2012 are depicted in Fig. 1.

## Main findings

As an initial step in our analyses, we compared pro-ED terms search volumes for the month prior to and at the month of news coverage involving Lady Gaga’s public ED disclosure. Findings indicated that there was a modest but non-significant increase in pro-ED terms search volumes from January 2012 (*M* = 38.50; *SD* = 14.98) to February 2012 (*M* = 45.83; *SD* = 22.21), *p* >. 05.

Next, we compared pro-ED search volumes for the month prior to and after publication of these news stories. Here, there was a statistically significant increase in pro-ED search volumes from January 2012 (*M* = 38.50; SD = 14.98) to March 2012 (*M* = 76.17; *SD* = 15.34), *t*(5) = 9.14; *p* < .05. Cohen’s *d* was 3.74, indicating a large effect size. On average, the search volumes increased by 37 % with term *pro ana websites* exhibiting the largest increase (52 %).

Finally, we compared pro-ED search volumes for the month at the time of and after publication of these news stories. Here, there was a significant increase in pro-ED search term volumes from February 2012 (*M* = 45.83; *SD* = 22.21) to March 2012 (*M* = 76.17; *SD* = 15.34), *t*(5) = 4.51; *p* <. 05. Cohen’s *d* was 2.03, indicating a large effect size. On average, the search volumes increased by 30 % per term with *pro ana websites* again exhibiting the largest increase (52 %).

**Fig. 2:**
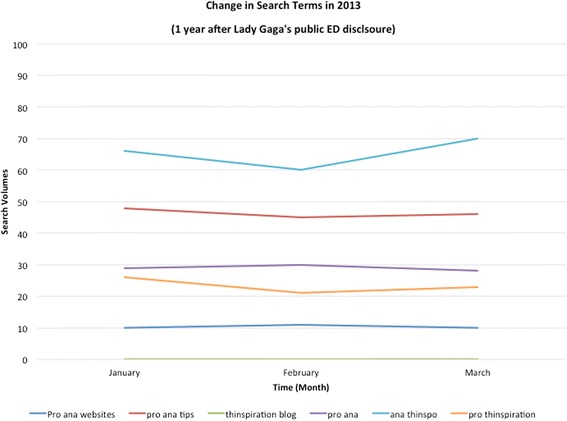
Search volumes for pro-ED key words via Google from January to March 2013. Notes: Values represent normalized rather than absolute values of the number of online pro-ED searches 1 year following Lady Gaga’s public ED disclosure via news media

To determine if the above findings were an artefact of the time of year, we examined the same search terms over the same time frame one year later. As shown in Fig. 2, there was no substantive change in pro-ED search patterns at this time. Paired t-tests comparing mean pro-ED search volumes between any months of interest (January, February, March) confirmed that changes were non-significant (*p* > .05). Finally, to examine if there were changes in pro-ED search patterns following stories involving ED disclosures of lower-profile celebrities, we looked at patterns of the next three celebrities based on social media popularity. As depicted in Table 1, the increases observed following Lady Gaga’s story were not observed for these stories.^2^ Moreover, paired t-tests confirmed that pro-ED search volume changes for these lower profile celebrities were non-significant, *p* > .05.

**Table 1:** Mean search volumes for pro-ED search terms following public ED disclosures of top 4 celebrities based on social media following

Celebrity	Month *Before* ED Disclosure Publicized	Month *When* ED Disclosure Publicized	Month *After* ED Disclosure Publicized
Lady Gaga	38.50	45.83	76.17
Demi Lovato	42.33	38.83	37.67
Kesha	27.00	33.50	28.83
Snooki	49.33	50.00	59.00

Notes: Values presented represent average standardized search volumes derived from Google Trends across the following search terms: *Pro ana website, Pro Ana, Pro ana tips, Ana Thinspo, Thinspiration blog, Pro thinspiration.* The 4 celebrities presented had the largest following on social media, indexed by total number of likes on Facebook and followers on Twitter. *ED* eating disorder

## Discussion

The current study was conducted to examine whether media coverage of celebrity ED disclosures corresponded with changes in Google searches for pro-ED terms. Findings suggest that pro-ED searches *may* increase soon after media coverage of high-profile celebrities who discuss past struggles with EDs. In the present study we found that in the month immediately following publication of one such story (i.e., Lady Gaga’s) there was a significant increase in the search volumes for pro-ED search terms when compared to both the month before the story was published and the month during which the story was published. This was not observed for the same period of time in the subsequent year. An important caveat to the trend observed is that the potential change in pro-ED search patterns might be circumscribed to particularly high-profile celebrity stories versus media coverage of celebrity ED disclosures in general.

The current findings build on research demonstrating that media coverage may inadvertently influence potentially harmful search trends online [7] by suggesting that a similar effect may occur when high-profile celebrity ED disclosures are covered. Our findings also suggest that pro-ED search terms identified in past work [2] may be especially salient in this regard. Indeed, the terms used in this study have been identified as associating with more pernicious pro-ED material. As a growing body of research has shown that access to pro-ED material online may contribute to offline ED pathology [1, 3], this is worrisome.

As with any study, the present findings should be interpreted within the context of several limitations. We cannot assume a causal relation between media story coverage of high-profile celebrity ED disclosures and Google search patterns for pro-ED content. Additionally, we do not have access to data about *who* conducted the Google searches reported and the impact (if any) these searches have on body image or ED behaviour. We also acknowledge that not all news stories of celebrity eating disorder disclosures yielded changes in pro-ED searches; indeed, this was only observed in the case of a higher-profile celebrity. Thus, the trend observed in the current study may only apply to particular celebrity stories versus *any* celebrity story. Finally, given the nature of data obtained, and because we were looking at individual search terms (versus an aggregate of multiple terms), we were limited in the types of analyses we could perform.

While there is certainly merit in reporting celebrity ED disclosures (e.g., help-seeking encouragement, de-stigmatization of EDs), some reports may evoke increases in pro-ED search patterns. Given the link between exposure to pro-ED content and ED behaviour [1–3], it seems prudent to intervene *prior to the point of access* - that is, before individuals access pro-ED websites. Hence, provision of recovery-oriented ED resources in search results following pro-ED searches may be warranted, similar to what is presently implemented for suicide-related searches on Google [6]. Likewise, providing pro-recovery resources within the reported news stories may be needed. Research-informed and supportive online ED resources are recommended in such efforts.

## Footnotes

1 Data were determined to have a normal distribution using the Shapiro-Wilk Statistic (W = .96; p > .05) prior to computing paired t-tests.

2 Overall, the changes in pro-ED search volumes following Lady Gaga’s news story were not observed for the other 3 celebrities depicted in Table 1. A similar pattern emerged for other lower-profile celebrity news stories not reported here.
